# The STRING database in 2023: protein–protein association networks and functional enrichment analyses for any sequenced genome of interest

**DOI:** 10.1093/nar/gkac1000

**Published:** 2022-11-12

**Authors:** Damian Szklarczyk, Rebecca Kirsch, Mikaela Koutrouli, Katerina Nastou, Farrokh Mehryary, Radja Hachilif, Annika L Gable, Tao Fang, Nadezhda T Doncheva, Sampo Pyysalo, Peer Bork, Lars J Jensen, Christian von Mering

**Affiliations:** Department of Molecular Life Sciences, University of Zurich, 8057 Zurich, Switzerland; SIB Swiss Institute of Bioinformatics, 1015 Lausanne, Switzerland; Novo Nordisk Foundation Center for Protein Research, University of Copenhagen, 2200 Copenhagen N, Denmark; Novo Nordisk Foundation Center for Protein Research, University of Copenhagen, 2200 Copenhagen N, Denmark; Novo Nordisk Foundation Center for Protein Research, University of Copenhagen, 2200 Copenhagen N, Denmark; TurkuNLP lab, Department of Computing, University of Turku, 20014 Turku, Finland; Department of Molecular Life Sciences, University of Zurich, 8057 Zurich, Switzerland; SIB Swiss Institute of Bioinformatics, 1015 Lausanne, Switzerland; Department of Molecular Life Sciences, University of Zurich, 8057 Zurich, Switzerland; SIB Swiss Institute of Bioinformatics, 1015 Lausanne, Switzerland; Department of Molecular Life Sciences, University of Zurich, 8057 Zurich, Switzerland; SIB Swiss Institute of Bioinformatics, 1015 Lausanne, Switzerland; Novo Nordisk Foundation Center for Protein Research, University of Copenhagen, 2200 Copenhagen N, Denmark; TurkuNLP lab, Department of Computing, University of Turku, 20014 Turku, Finland; Structural and Computational Biology Unit, European Molecular Biology Laboratory, 69117 Heidelberg, Germany; Yonsei Frontier Lab (YFL), Yonsei University, Seoul 03722, South Korea; Max Delbrück Centre for Molecular Medicine, 13125 Berlin, Germany; Department of Bioinformatics, Biozentrum, University of Würzburg, 97074 Würzburg, Germany; Novo Nordisk Foundation Center for Protein Research, University of Copenhagen, 2200 Copenhagen N, Denmark; Department of Molecular Life Sciences, University of Zurich, 8057 Zurich, Switzerland; SIB Swiss Institute of Bioinformatics, 1015 Lausanne, Switzerland

## Abstract

Much of the complexity within cells arises from functional and regulatory interactions among proteins. The core of these interactions is increasingly known, but novel interactions continue to be discovered, and the information remains scattered across different database resources, experimental modalities and levels of mechanistic detail. The STRING database (https://string-db.org/) systematically collects and integrates protein–protein interactions—both physical interactions as well as functional associations. The data originate from a number of sources: automated text mining of the scientific literature, computational interaction predictions from co-expression, conserved genomic context, databases of interaction experiments and known complexes/pathways from curated sources. All of these interactions are critically assessed, scored, and subsequently automatically transferred to less well-studied organisms using hierarchical orthology information. The data can be accessed via the website, but also programmatically and via bulk downloads. The most recent developments in STRING (version 12.0) are: (i) it is now possible to create, browse and analyze a full interaction network for any novel genome of interest, by submitting its complement of encoded proteins, (ii) the co-expression channel now uses variational auto-encoders to predict interactions, and it covers two new sources, single-cell RNA-seq and experimental proteomics data and (iii) the confidence in each experimentally derived interaction is now estimated based on the detection method used, and communicated to the user in the web-interface. Furthermore, STRING continues to enhance its facilities for functional enrichment analysis, which are now fully available also for user-submitted genomes.

## INTRODUCTION

A dense network of functional connections among proteins has evolved to support cellular life, forming a multitude of pathways, protein complexes and cellular structures (1,2). Recent developments have further improved our ability to unravel this connectivity, through techniques such as high-throughput genetic screens (3–5), systematic co-fractionation of proteomes (6,7), *in-vivo* proteome-wide cross-linking of proteins (8–10), and deep learning-based computational prediction frameworks such as AlphaFold (11,12). These efforts complement earlier results based on focused, small-scale laboratory studies, yeast two-hybrid screens, affinity purifications, co-crystallization and computational prediction algorithms (reviewed in (13)). Together, the many approaches have begun to reveal a large part of the interaction landscape of cellular proteins, but they each have strengths and weaknesses, including potential biases, false negatives and noise. Furthermore, the resulting interaction data is scattered over a number of online resources, and available in varying namespaces and data-formats as well as varying levels of detail.

Given this, a number of meta-resources dedicated to data integration in the protein network context have been developed. These resources aim to collect and critically assess protein–protein functional linkage data, integrate it, connect it to previous knowledge, and allow users to browse, compare and retrieve organism-wide protein networks. Among frequently used and actively maintained frameworks are ConsensusPathDB (14), FunCoup (15), GeneMANIA (16), HumanBase (17), HumanNet (18), IID (19) and STRING (20–22). Within this group, STRING places its focus on comprehensiveness and ease of use—it covers >10 000 organisms, draws from a wide diversity of data sources including text mining and computational predictions, and offers many intuitive interface features including personalization, enrichment detection and programmatic access.

Researchers employ protein network meta-resources for a wide variety of purposes, broadly falling into three categories: (i) facilitating individual, small-scale molecular discoveries, (ii) facilitating large-scale data analysis and (iii) contributing to new methodologies and workflows. With respect to STRING, it has, for example, proven useful in interpreting and reducing newly acquired genetic screening datasets—these are sometimes noisy and unwieldy, and STRING can be used to distill such data into more manageable sets of observations and hypotheses. As a case in point, consider three recent screens for SARS-CoV-2 human host factors (23–25). All three studies used STRING to interpret their initial, raw lists of screening hits—placing them into network contexts and searching them for functionally enriched processes/pathways. STRING and its competitors are also often used as data providers in novel methodologies and computational resources, be it new databases, new algorithms, or community-wide competitions. As an example, consider two recent uses of STRING networks in deep-learning frameworks (26,27). Both studies use deep learning to predict protein function (i.e. Gene Ontology terms), based on amino acid sequences and protein–protein interaction network topologies derived from STRING.

Here, we provide an update on the current features of the STRING database and describe some novel developments in more detail. The latter include a complete redesign of the co-expression based interaction prediction pipeline, newly exposed sub-scores for experimental dataset confidence, as well as novel facilities allowing users to upload and analyze any newly sequenced genome of interest.

## DATABASE CONTENT

The scope of protein–protein links in STRING is that of a ‘functional association’ (28–30)—proteins are considered to be associated when there is evidence suggesting an evolved, specific functional partnership between the two. This definition includes proteins that are physically associated to each other in a protein complex or in a transient interaction, but also proteins that are more indirectly associated: they may work towards a common goal in a metabolic or signaling pathway, may regulate each other through intermediaries, or may jointly contribute to a common cellular structure. The granularity of what constitutes a ‘common function’ is not formally defined; it should not be understood too broadly, however, and operationally it roughly corresponds to the specificity of pathways ‘maps’ or ‘diagrams’ in knowledgebases such as KEGG (31) or Reactome (32). It should be noted that the definition of a functional assocation can include proteins that act antagonistically to each other, albeit in the same overall pathway.

All protein–protein association evidence in STRING is assessed and quantified, and its correspondence to the above definition is benchmarked against common membership in KEGG pathway maps (excluding maps that are largely based on homology, such as ‘ABC transporters’). The result of this benchmarking/calibration is a STRING ‘confidence score’ for each association; confidence scores are scaled between zero and one, and correspond to the estimated likelihood of a given association being true, given the underlying evidence. Confidence scores are first computed separately per evidence type (see (33) for an example), and then integrated into a final, ‘combined’ confidence score. All evidence collected for a given protein pair contributes to the score, irrespective of the exact nature of these proteins in terms of alternative splicing isoforms or post-translational modifications; correspondingly, the interacting unit in STRING networks is the entire protein-coding locus, represented by its most canonical protein product (34).

All confidence scores in STRING are pre-computed and freely available for download, under a Creative Commons Attribution license (CC BY 4.0). The various evidence types are grouped into seven distinct ‘evidence channels’, with separate sub-scores available for each channel. These channels can also be individually viewed on the STRING user interface, together with their underlying evidence, and can be enabled or disabled separately as desired. Of the seven channels, the first three (‘neighborhood’, ‘fusion’ and ‘co-occurrence’) are dealing with association evidence that can be gleaned from genome sequences alone. These so-called ‘genomic context’ channels (reviewed in (35,36)) are based on detecting evolutionary constraints arising from functional gene-gene partnerships, and are best applicable in prokaryotic genomes. Another channel (‘co-expression’) is dealing with functional genomics measurements (transcripts or proteins) across a multitude of conditions, searching for evidence of common expression regulation (see also below). Next, the ‘experiments’ channel deals with laboratory experiments that were conducted with the expressed goal to uncover protein–protein association evidence. They are imported from primary database repositories: BioGRID (37), DIP (38), PDB (39), as well as IntAct and its partner databases in the IMEx consortium (40). The final two evidence channels are concerned with protein–protein associations that are already known. The ‘database’ channel imports well-established knowledge (‘textbook knowledge’) about protein complexes, pathways and other functional connections from dedicated knowledge resources: KEGG (31), Reactome (32), MetaCyc (41), EBI Complex Portal (42), and Gene Ontology Complexes (43). Lastly, the ‘textmining’ channel is the result of parsing full-text articles from the PMC Open Access Subset (up to April 2022), PubMed abstracts (up to August 2022), as well as summary texts from OMIM (44) and SGD (45) entry descriptions. These texts are all parsed for co-mentions of protein pairs and assessed against the frequencies of all separate mentions of the respective proteins, as described in (46). On top of functional associations, the three latter channels (i.e. experiments, database and textmining) provide also the interactions for the physical sub-network of STRING. The calculation of the confidence scores for the protein pairs in this network differs from that of the functional association network and is described in detail in (22).

The deep learning-based relation extraction text mining model has received significant upgrades in the current version. Specifically, the language representation model that we use has changed from BioBERT (47) to a biomedical RoBERTa-large model (48), which has already shown better performance in relation extraction tasks (49). Moreover, the model can now detect physical interactions that span across the boundaries of a single sentence. This is made possible mainly due to the two orders of magnitude increase of manually annotated relations in the training set (from 6145 to 243 831), which led to the addition of many cross-sentence pairs during training, thus allowing the model to learn to detect such associations. These changes in the deep learning model, in combination with the increase in the size of the literature corpus compared to the previous STRING version, have led to a two-fold increase in the number of physical protein–protein interactions above the low, medium, and high confidence score cut-offs in the text mining channel (Table 1).

**Table 1.** Counts and relative frequencies of physically interacting protein pairs obtained via text mining. Aggregating across all organisms in STRING, the table shows counts for various frequently used score cutoff levels, for both STRING version 11.5 and STRING version 12.0. The lowest score cut-off has been used to determine what constitutes 100% for each dataset

score	Number of pairs in v. 11.5	Number of pairs in v. 12.0	Frequency of pairs above score cut-off in v. 11.5 (%)	Frequency of pairs above score cut-off in v. 12.0 (%)
0.15	253 626	401 976	100.0	100.0
0.4	70 148	143 591	27.6	35.7
0.7	22 349	45 981	8.8	11.4
0.9	0	21 689	0	5.4

All protein–protein associations assembled for the STRING database are then transferred across organisms, where applicable, based on orthology relationships with the assumption that orthologs of associated proteins are likewise associated (‘interologs’, (50)). For this, hierarchical orthology relationships, at various levels of taxonomic resolution, are imported from the eggNOG database (51). After interolog transfer and the final combined score integration, the resulting protein association networks can be accessed in a number of ways. Firstly, the interactive website of STRING allows browsing and searching, including evidence inspection via dedicated viewers. Users can also submit larger queries there, allowing for the construction of dedicated networks and for statistical searches for functional enrichments. Apart from the website, scientists can access STRING via a dedicated Cytoscape plugin (52), as well as via a Bioconductor package and a dedicated application programming interface (REST).

## USER-UPLOADED GENOMES

One of the unique features of STRING has always been its support for a large selection of non-model organisms: the current version of the database contains protein–protein interaction networks (and protein functional pathway annotations) for >10 000 distinct genomes. When selecting which genomes to include in a STRING update, key factors are the organism's research prominence, genome quality, and completeness. Subsequently, STRING utilizes genomes from authoritative sources only, including Ensembl (53), UniProtKB Reference Proteomes (54) and the ‘representative genomes’ set in the proGenomes database (55).

However, new genomes are sequenced and assembled daily, and existing genomes are re-sequenced or re-annotated; overall, the number of taxonomically distinct species that are being sequenced has been doubling roughly every 3 years (56). The UniProtKB database is updated with the new proteomes on an eight-week cycle, and projects like Ensembl Rapid Release (53) aim to provide annotation for the newly released genomes on a 2-week cycle. For STRING, such frequent update cycles would require heavy resources and may create complexities with data reproducibility/data archiving when the new updates supersede older datasets. On the other hand, a slow update cycle implies that the database will not incorporate newly sequenced genomes or any improvements to the gene sets of existing genomes.

**Figure 1.**
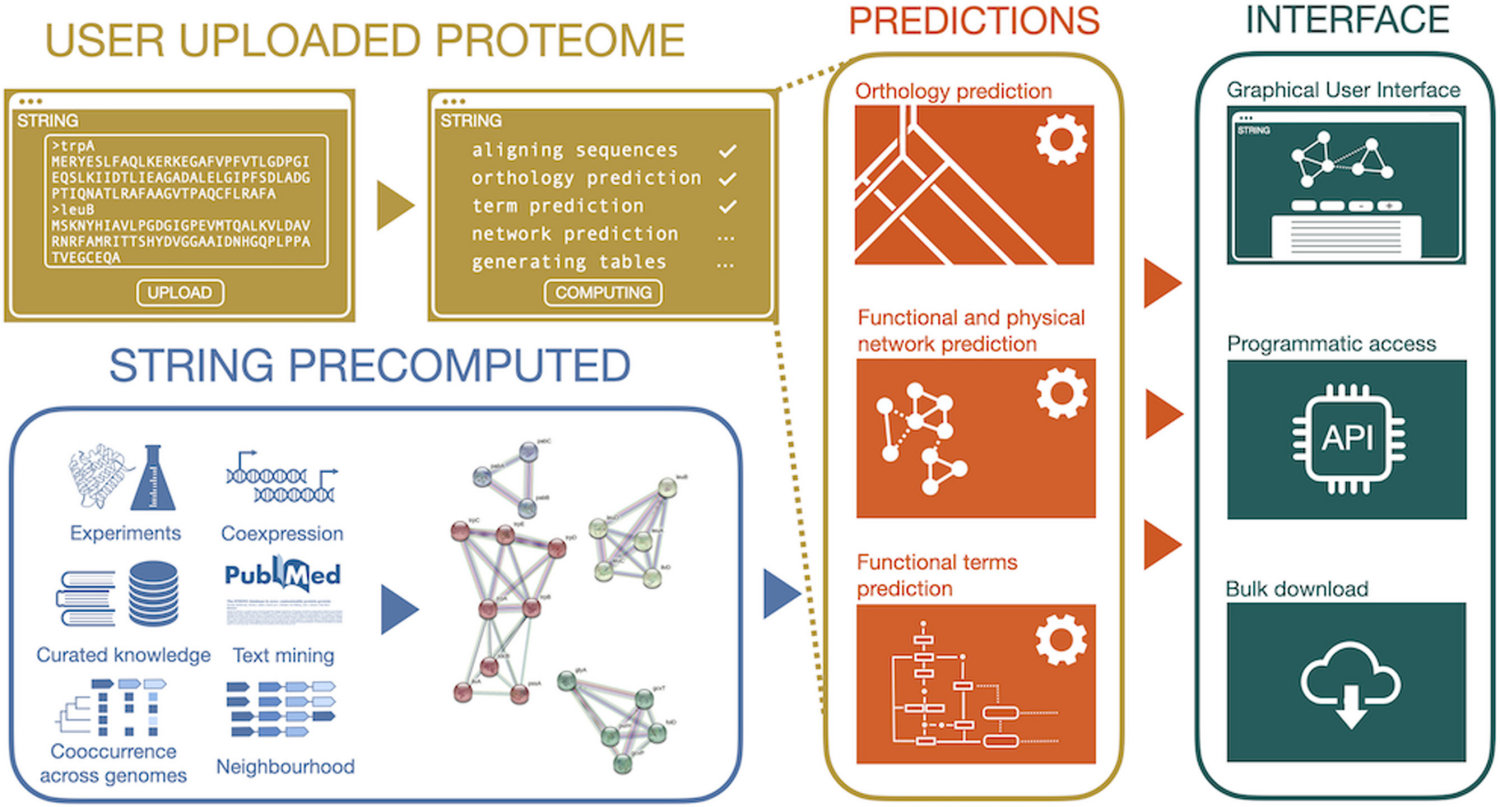
Extending STRING with user-submitted genomes. Submitted genomes are first searched against the existing STRING genomes, and orthology is used to transfer all relevant information (interactions, annotations) from closely related organisms. The submitted genomes then become available on the web interface, via the programmatic Application Programming Interface (API), and for bulk downloads.

To improve this situation, STRING now allows its users to upload any fully sequenced proteome (including those that are already part of the database), enabling them to browse and query the predicted protein–protein interactions in an identical manner to the genomes already natively covered by the STRING database. This includes access to the evidence viewers, homology viewer, network clustering methods, gene set enrichment analysis, bulk download, and REST API access. The outline of the proteome annotation pipeline is shown in Figure 1. The procedure for uploading a new proteome begins by choosing ‘Annotate your proteome’ on the STRING input page, after which the user is guided through a few simple steps of the process. All that is required for the submission is a simple FASTA-formatted proteome, as well as the taxonomic name of the species or clade that the uploaded proteome belongs to. Along with the protein sequences from the file, if provided, STRING will extract from the FASTA definition lines (headers) any standard gene names, identifiers, and free-text protein descriptions; these will later be searchable from the input page and used throughout the webpage. For this, STRING automatically recognizes several formats of FASTA headers including those from RefSeq (57), UniProtKB (54) and Ensembl (53), and checks for any apparent errors such as duplicate sequence identifiers. After the proteome is uploaded, STRING directly aligns the sequences to all sequences in its database using DIAMOND (58) (with the --iterate option). Each protein is then assigned to its orthologs via the hierarchical orthology database eggNOG (51) based on its highest scoring alignment (best hit). The taxonomic level at which the protein is placed in the group hierarchy is defined by the last common ancestor between the user-specified taxon and the taxon of the best-scoring hit. If a protein cannot be placed via the hierarchical orthology groups, it is considered a direct one-to-one ortholog with its best-scoring hit. As the user can submit a proteome of an organism already included in the database, the proteins of the matching proteomes will then directly map to each other, without a need for mapping to any of the existing orthologous groups.

The network prediction based on orthology is then performed as described previously (46). In parallel with the network prediction, STRING also attempts to assign the submitted proteins to their corresponding pathways and functional subsystems, as imported for STRING from the three Gene Ontology branches (Biological process, Molecular Function and Cellular Component) (43), KEGG pathways (31), UniProtKB Keywords (54), COMPARTMENTS (59) and TISSUES (60). This assignment is done based on the consensus of the pathway annotations of the pre-existing STRING proteins in the most specific orthologous group that a new protein is assigned to. If these pre-existing proteins carry no pathway annotation, the consensus is attempted in the next-higher, parental orthologous group in the orthology hierarchy; this is repeated until it is successful or until the root level is reached. STRING does not assign pathways or functional subsystems outside their previously known taxonomic scope. A pathway's scope is defined by the last common ancestor of all organisms that have in the past been annotated with this pathway. At the end of the computation process, all predictions are automatically uploaded to the internal STRING database, bulk download files are generated, and the user is given a unique STRING proteome identifier. This identifier functions as an organism name on the input page, making the newly submitted genome browsable and searchable. Submitters can share this identifier with other users, but if they choose not to, the submitted genomes remain private.

## IMPROVED CO-EXPRESSION ANALYSIS

The degree of co-expression between RNAs (or between proteins) across different conditions provides an essential insight into the functional protein–protein interaction network of a cell (61–64). STRING collects gene expression evidence from RNA expression arrays and RNA-Seq datasets processed by the GEO database (65) as well as co-regulation evidence from the ProteomeHD database (7). In version 12.0 of STRING, the co-expression network is being extended with evidence from two novel sources: single-cell RNA-Seq data from the Human Protein Atlas (66) and proteomics datasets from the PRIDE database (67).

The expression data tends to be sparse, high-dimensional and highly redundant. These attributes decrease the performance of correlations using Pearson Correlation Coefficient. For previous versions of STRING we have reduced the redundancy by removing highly correlated expression matrices before correlating the gene expression which, in turn, increased the recovery of the functional associations derived from these matrices. However, this did not fully address all of the challenges of such datasets.

**Figure 2.**
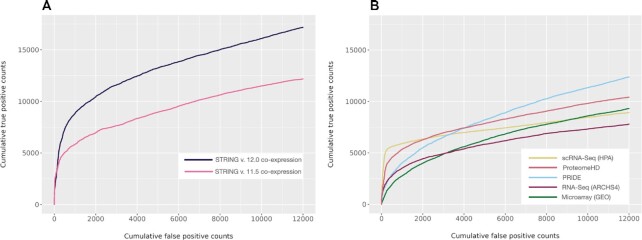
Improved interaction prediction based on co-expression. Interaction scores based on co-expression have been ranked and benchmarked against common KEGG pathway membership as ground truth. (**A**) Performance comparison of co-expression network between STRING version 11.5 and STRING version 12.0. (**B**) Overview of the performance of all expression datasets contributing to the STRING version 12.0 co-expression channel.

To address that, in the latest version we have utilized a novel method called FAVA (Functional Associations using Variational Autoencoders) (68) to build STRING’s co-expression network. This deep-learning model reduces the dimensionality of the data into lower-dimensional latent spaces using variational autoencoders (VAE). The benefit of encoding the matrices into fewer dimensions is 2-fold: it reduces the overall sparsity of the data and limits redundancy by essentially compressing the data. The predictions obtained from all the sources are combined in a probabilistic fashion (46) and the resulting scores are recalibrated to negate the effect that the non-independence of the sources has on the STRING network. New methods together with the additional sources result in significant improvement in the performance of STRING version 12.0 over STRING version 11.5 co-expression network (Figure 2A). The performance of each individual source contributing to the combined network is shown in Figure 2B. In addition, the STRING interface now reports the associated fractional score and the source of each piece of evidence in the co-expression network.

RNA-Seq datasets have increased sensitivity for genes exhibiting low levels of expression (69,70). This combined with improved performance and the high-throughput nature of the experimental data reduces the inherent literature bias of the STRING network.

## EXPERIMENT-LEVEL CONFIDENCE SCORING

**Figure 3.**
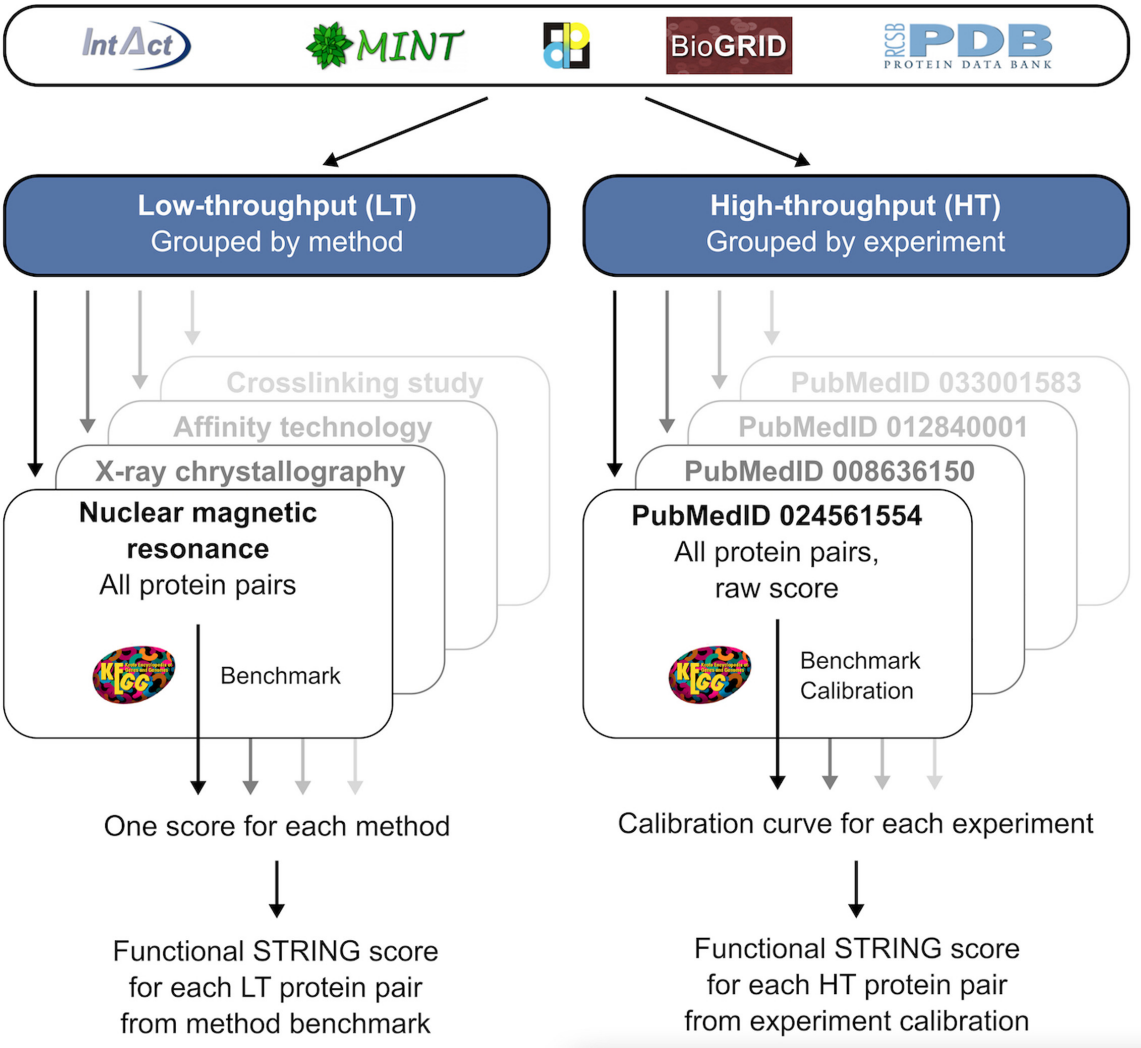
Processing and scoring of experimental interaction evidence. Experimental evidence is retrieved from several public databases. Protein pairs from low-throughput (LT) experiments are grouped by detection method and pairs coming from high-throughput (HT) experiments are grouped by experiment. Within each group, pairs are benchmarked against the KEGG pathway database to assess the confidence of identifying functional associations. All LT pairs are assigned the benchmark score derived for the particular detection method. HT pairs are scored based on calibration on the experiment level.

For the experiments channel, STRING integrates pairwise experimental interaction evidence from BioGRID, IntAct, MINT, and others ((37–40), see Figure 3). Each reported protein pair supported by a specific publication is considered an independent piece of evidence and scored individually. During import, duplicate records from different sources are removed. Each ‘experiment’, defined as all protein–protein pairs supported by a common PubMed identifier and a given detection method, is classified as a high-throughput (HT) experiment if at least 25 unique interactions are reported, otherwise as a low-throughput (LT) experiment.

For interactions detected by HT experiments, benchmarking against KEGG molecular pathway maps and scoring is performed separately for each experiment: each pair is assigned a raw score based on the number of shared and non-shared interactors for both proteins within this experiment. The more shared and the fewer non-shared interactors, the higher the raw quality score of the protein pair from this experiment. Based on the raw score, all pairs detected in the experiment are ranked and benchmarked against KEGG. A typical STRING calibration function is derived for each HT experiment, which is then used to assign a functional association score to each protein pair identified in the experiment (33).

For the benchmarking and scoring of LT experiments, STRING makes use of the Molecular Interaction Controlled Vocabulary (PSI-MI CV, https://www.ebi.ac.uk/ols/ontologies/mi, (71)). Specifically, STRING considers the methods annotated as ‘experimental interaction detection’ (MI:0045) and its children but excludes those that are inferred by the author/curator (MI:0363 and MI:0364) or predicted (MI:0063). Among the included methods, evidence generated by ‘genetic interference’ methods (MI:0254) is considered as strictly functional associations, while all other methods provide physical interaction evidence, which inherently is evidence for functional associations as well. Protein pairs are grouped by experimental interaction detection method, to obtain groups that are large enough for benchmarking. Because protein pairs detected by LT experiments tend to focus on specific pathways or known proteins, no curve-based score calibration is done. Instead, STRING directly translates the overall true-positive rate of each interaction detection method into the confidence score for that method. Each protein pair detected in an LT experiment by this method is then assigned the corresponding method's score. By aggregating all scores for a particular protein pair across experiments, one experimental functional association score for that pair is derived.

**Figure 4.**
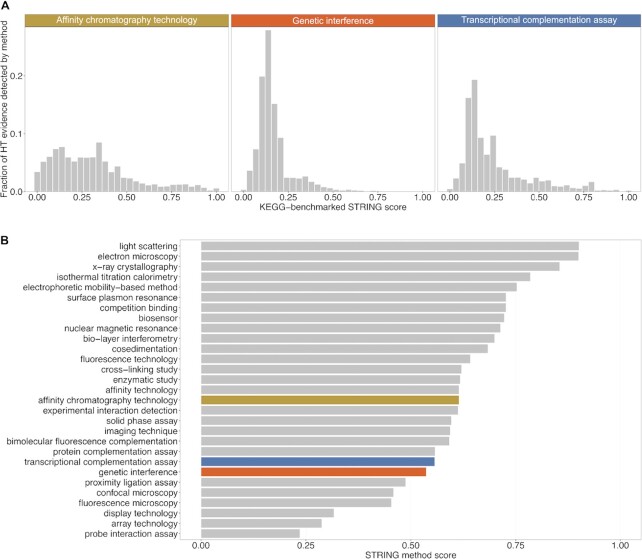
Reliability estimates for protein–protein interaction detection assays. The top three most prolific experimental interaction assay types are shown, ranked by the number of protein pairs to which they contribute in STRING. Benchmarked on KEGG pathways, they yield (on average) lower confidence scores when derived from the high-throughput assays (**A**), in contrast, for the equivalent low-throughput experiments (shown in color), the predicted confidence is substantially higher (**B**). The distributions shown in (A) encompass all HT interactions of a given assay type (for simplicity); in the actual scoring computations in STRING, each HT interaction datasets is scored separately.

The highest number of experimentally detected associations has been derived by affinity chromatography technologies (40%), followed by genetic inference (34%) and transcriptional complementation assays (11%). Out of these, 82% came from HT experiments. The associations derived from these three HT experiments, on average, place below or around a confidence score of 0.25 (Figure 4A), while, in comparison, the LT experiments for the same methods score more than twice as high with a medium confidence score of around 0.6 (Figure 4B). The top three methods by confidence score for LT-derived associations are assays determining the 3D structure of protein complexes, which not surprisingly are excellent predictors of functional and physical protein–protein interactions.

In STRING’s ‘experimental’ evidence viewer, the user can now better appreciate the reliability of each experimental prediction, as the individual confidence for every HT and LT dataset is now communicated to the user in a form of three-tier confidence grade (‘high’, ‘medium’ and ‘exploratory’).

## OUTLOOK

Version 12.0 of STRING covers a phylogenetically diverse collection of 12 535 high-quality genomes. Beyond these, the system will record which genomes are frequently submitted by users—and these will then be prioritized for inclusion into subsequent releases. In addition, the results of an ongoing online user survey will be analyzed (350 users have already participated). This way, STRING will keep concentrating its resources on those areas that are of most interest to its users.

## DATA AVAILABILITY

STRING is freely available, under a Creative Commons Attribution license (CC BY 4.0).
